# Elevated serum granulocyte-macrophage colony-stimulating factor levels during radiotherapy predict favorable outcomes in lung and esophageal cancer

**DOI:** 10.18632/oncotarget.13202

**Published:** 2016-11-08

**Authors:** Guodong Deng, Pingping Hu, Jingxin Zhang, Qiqi Liu, Ning Liang, Jian Xie, Lili Qiao, Hui Luo, Deguo Xu, Fengjun Liu, Xinshuang Yu, Zhen liu, Yajuan Lv, Jiandong Zhang

**Affiliations:** ^1^ Department of Radiation Oncology, Qianfoshan Hospital, Shandong University, Jinan 250014, PR China; ^2^ Division of Oncology, Department of Graduate, Weifang Medical College, Weifang 261053, PR China; ^3^ Department of Oncology, The Fifth Peoples' Hospital of Jinan, Jinan 250022, PR China; ^4^ Department of Radiation Oncology, Henan Cancer Hospital Affiliated to Zhengzhou University, Zhengzhou University, Zhengzhou 450001, Henan, China

**Keywords:** granulocyte-macrophage colony-stimulating factor, interferon-γ, radiotherapy, cancer prognosis

## Abstract

The combination of exogenous granulocyte-macrophage colony-stimulating factor (GM-CSF) with radiotherapy (RT) has been demonstrated to strengthen the antitumor immune response. We hypothesized that the variation of GM-CSF during RT was correlated with cancer prognosis. We measured serum levels of GM-CSF and interferon-*γ* (IFN-*γ*) before and during RT in 126 unresectable lung and esophageal cancer patients and performed survival analyses. Upregulated GM-CSF levels during RT correlated with longer overall survival (OS) and progression-free survival (PFS). On the other hand, no difference in OS or PFS was seen at different IFN-*γ* levels. However, the “integrated factor”, computed as the combination of high pre-RT IFN-*γ* levels and upregulated GM-CSF, correlated with prolonged OS and PFS. Multivariate analyses revealed that GM-CSF levels and the integrated factor were both stronger independent prognostic factors than disease stage. These data demonstrate that GM-CSF levels during RT can be used as a prognostic biomarker for lung and esophageal cancer.

## INTRODUCTION

Lung cancer (LC) and esophageal cancer (EC) are both the most frequently diagnosed cancers and the leading causes of cancer death worldwide [1]. In spite of recent progress in diagnosis and treatment, recurrence and mortality rates remain high for LC and EC [2, 3]. Such poor prognosis might be at least partially attributable to the lack of effective prognostic factors to inform on optimal therapeutic choices. Effective novel prognostic biomarkers could improve prognosis for LC and EC patients.

Granulocyte macrophage colony-stimulating factor (GM-CSF) is a glycoprotein mainly secreted by immune cells, fibroblasts, endothelial cells and some tumor cells [4]. Conventionally, it is a common growth factor for blood cells, used in myelosuppression resulting from chemoradiotherapy. Recent evidence suggests that GM-CSF might can be used to treat malignancies by enhancing the antitumor immune response [5–7]. Furthermore, both experimental and clinical applications of GM-CSF or GM-CSF-encoded tumor vaccine as cancer therapies showed promising results [8–11].

Radiotherapy (RT) is an established antitumor treatment. In addition to its cytotoxic effects, it also contributes to the antitumor immune response by promoting the release of tumor cell antigens such as heat shock proteins and high mobility group box 1 after tumor cell necrosis [5, 7]. Furthermore, tumor infiltration of macrophages was also observed after RT both in animal models and in human samples [12, 13]. When combined with GM-CSF therapy, RT induced regression of tumors distant from the irradiated site, which is defined as abscopal effects [14, 15]. This suggests that the combination of GM-CSF with RT yields synergistic therapeutic effects. An ongoing clinical trial is evaluating the efficacy of this combined therapy for late stage lung cancer patients [16]. However, few studies have investigated the effect of endogenous GM-CSF levels during RT on survival. In the current study, we hypothesized that GM-CSF levels during RT might affect prognosis. Therefore, we measured the variation of GM-CSF levels in serum during RT of unresectable LC and EC patients. We also calculated the correlations of GM-CSF levels with clinicopathological features, and explored simultaneous circulating immune cells. Lastly, to explain the prognostic role of GM-CSF for irradiated patients according to the antitumor immune cycle [5], we also measured the levels of serum IFN-*γ*.

## RESULTS

### Patients

A total of 126 patients (96 men and 30 women) were enrolled in this study, including 72 (57.1%) in the LC group and 54 (42.9%) in the EC group. The median age was 63 for the LC group (range: 26-81), 65 for the EC group (range: 43-86), and 64 for entire population group (range: 26-86). In the LC group, the number of patients with squamous cell carcinoma (SCC), adenocarcinoma (AC) and small cell lung cancer (SCLC) were 32, 28 and 9, respectively. Almost all EC patients were SCC histology type. There were 26 patients in the LC group and 32 in the EC group with stage II-III cancer, and 46 LC patients and 22 EC patients with stage IV cancer. In the LC group, 45 accepted concurrent chemoradiothearpy and 27 accepted radiotherapy only. On the other hand, for EC groups these numbers were 37 and 17, respectively. Baseline patient features are listed in Table 1.

**Table 1:** Baseline features of patients separated by GM-CSF levels during RT

Features	GM-CSF levels					
	Lung cancer			Esophageal cancer		
	Upregulated N (%)	Downregulated N (%)	Total N (%)	Upregulated N (%)	Downregulated N (%)	Total N (%)
**Age**						
Below median	15 (20.8%)	23 (31.9%)	38 (52.8%)	11 (20.4%)	14 (25.9%)	25 (46.3%)
Above median	19 (26.4%)	15 (20.8%)	34 (47.2%)	12 (22.2%)	17 (31.5%)	29 (53.7%)
**Gender**						
Male	26 (36.1%)	28 (38.9%)	54 (75.0%)	17 (31.5%)	25 (46.3%)	42 (77.8%)
Female	8 (11.1%)	10 (13.9%)	18 (25.0%)	6 (11.1%)	6 (11.1%)	12 (22.2%)
**Pathology**						
SCC	17(23.6%)	15(20.8%)	32(44.4%)	22(40.7%)	28(51.9%)	50(92.6%)
AC	11(15.3%)	17(23.6%)	28(38.9%)	0(0%)	0(0%)	0(0%)
SCLC	5(6.9%)	4(5.6%)	9(12.5%)	-	-	-
Others	1(1.4%)	2(2.8%)	3(4.2%)	1(1.9%)	3(5.6%)	4(7.4%)
**Stage**						
II-III	18 (25.0%)	28 (38.9%)	46 (63.9%)	9 (16.7%)	13 (24.1%)	22 (40.8%)
IV	16 (22.2%)	10 (13.9%)	26 (36.1%)	14 (25.9%)	18 (33.3%)	32 (59.2%)
**Treatment strategy**						
RT	5 (6.9%)	22 (30.6%)	27 (37.5%)	5 (9.3%)	12 (22.2%)	17 (31.5%)
CRT	29 (40.3%)	16 (22.2%)	45 (62.5%)	18 (33.3%)	19 (35.2%)	37 (68.5%)

Abbreviations: GM-CSF=granulocyte-macrophage colony stimulating factor; N=number; SCC=squamous cell carcinoma; AC=adenocarcinoma; SCLC=small cell lung cancer; RT=radiotherapy; CRT=chemoradiotherapy.

### Serum GM-CSF and IFN-*γ* levels

Pre- and during RT serum GM-CSF levels were 129 ± 31 pg/ml and 127 ± 30 pg/ml for LC group, 90 ± 20 pg/ml and 93 ± 18 pg/ml for EC group, no significant difference in GM-CSF levels were observed between pre- and during RT for these two groups (p=0.477 and 0.214). In total, 57 patients showed elevated GM-CSF levels during RT, 34 of these belonged to the LC group and 23 to the EC group. The median pre-RT IFN-*γ* level was 62 pg/ml for all patients, 60 pg/ml for the LC group and 62 pg/ml for the EC group. In total, 69 patients showed high IFN-*γ* levels during RT, 41 belonging to the LC group and 28 to the EC group. Of 57 patients with upregulated GM-CSF, 38 showed high IFN-*γ* levels. Of all 69 patients with downregulated GM-CSF, 38 showed low IFN-*γ* levels.

### Survival analysis

The median OS was 11 months for the entire population group, 11 months for the LC group and 12.5 months for the EC group. The median PFS was 7 months for all of the 3 groups. In all groups, patients with upregulated GM-CSF had longer OS and PFS than patients with downregulated GM-CSF (all p values <0.05, log-rank test).

**Figure 1:**
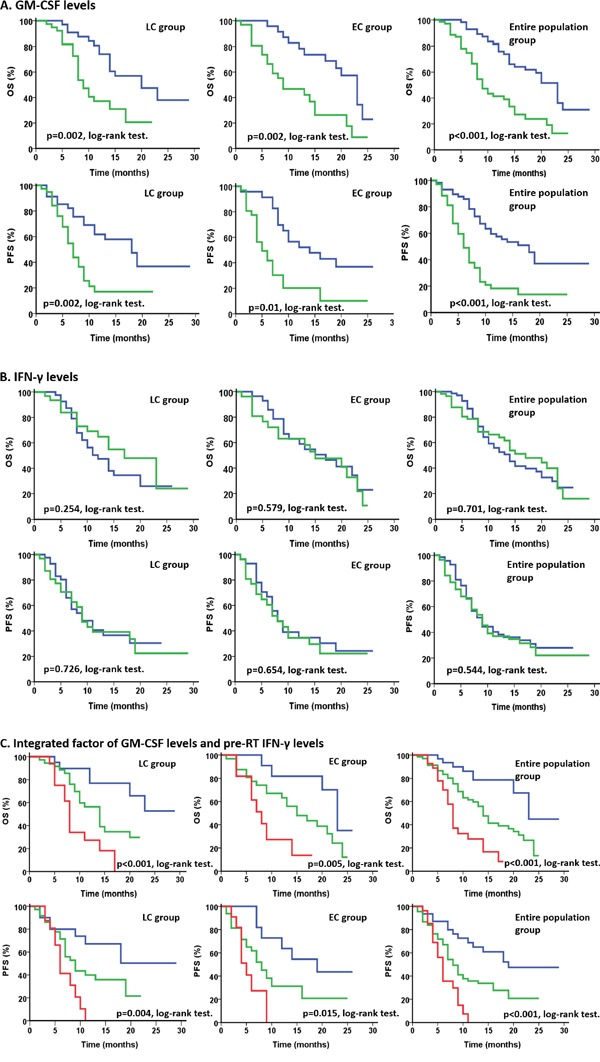
Survival curves for OS and PFS in all 3 groups **A.** Survival analysis according to GM-CSF levels during RT, with blue indicating upregulated GM-CSF levels and green denoting downregulated GM-CSF levels. Patients in the EC, LC, and entire population groups with upregulated GM-CSF levels had longer OS and PFS than patients with downregulated GM-CSF levels (all p<0.05, Log-rank test). **B.** Survival analysis according to IFN-*γ* levels during RT, with blue indicating upregulated IFN-*γ* levels and green denoting downregulated IFN-*γ* levels. No difference in OS and PFS was observed for patients in the LC, EC, and entire population groups, when separated by change in IFN-*γ* levels (all p>0.05, Log-rank test). **C.** Survival analysis by integration of GM-CSF levels during RT and pre-RT IFN-*γ* levels. Blue indicates upregulated GM-CSF levels during RT and high pre-RT IFN-*γ* levels, while green indicates downregulated GM-CSF levels during RT and low pre-RT IFN-*γ* levels. In the LC, EC, and entire population groups, patients with upregulated GM-CSF levels during RT and high pre-RT IFN-*γ* levels had the best prognosis while patients with downregulated GM-CSF levels during RT and low pre-RT IFN-*γ* levels had the worst prognosis (all p<0.05, Log-rank test).

Because IFN-*γ* is an important effector molecule secreted by cytotoxic T cells [7] and a previous study showed that GM-CSF and IFN-*γ* exert synergistic antitumor immune effects when combined [17], we performed survival analyses using IFN-*γ* levels. However, there was no difference between patient OS and PFS when separated by IFN-*γ* levels (all p>0.05, log-rank test). Subsequently, we performed survival analysis using the integrated factor of these two cytokines. Patients with upregulated GM-CSF and high pre-RT IFN-*γ* levels showed the best OS and PFS, while patients with downregulated GM-CSF and low pre-RT IFN-*γ* levels showed the worst OS and PFS (p <0.05 for all 3 groups). Survival curves are listed in Figure 1.

**Figure 2:**
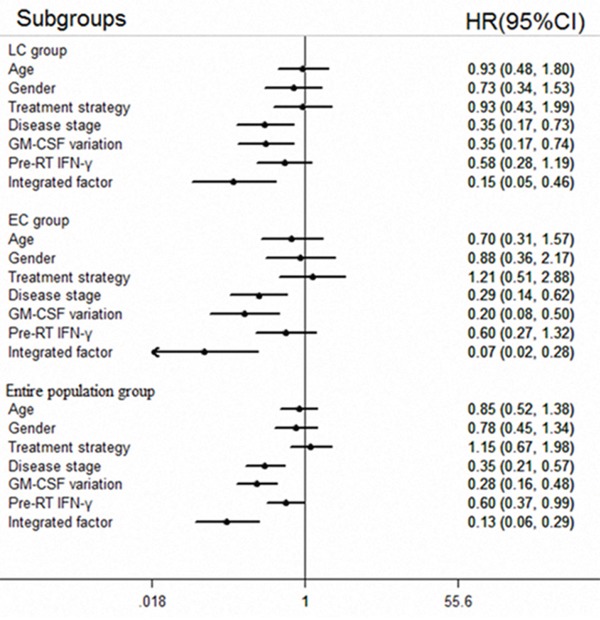
Forest plot for multivariate analysis The forest plot shows merged outcomes of multivariate survival analysis for the LC, EC, and entire population patient groups. Disease stage, GM-CSF levels and the integrated factor are independent prognostic factors. The integrated factor yielded the best prognostic value compared with disease stage and GM-CSF levels.

Multivariate analysis suggested that GM-CSF levels were an independent prognostic factor for all three groups (LC group: HR=0.355, p=0.006; EC group: HR=0.202, p<0.001; entire population group: HR=0.280, p <0.001). Disease stage was also an independent prognostic factor for all three groups (LC group: HR=0.348, p=0.006; EC group: HR=0.202, p<0.001; entire population group: HR=0.346, p <0.001). Furthermore, the integrated factor was also an independent prognostic factor for all three groups (LC group: HR=0.153, p=0.001; EC group: HR=0.071, p <0.001; entire population group: HR=0.128, p <0.001). On the other hand, pre-RT IFN-*γ* levels were an independent prognostic factor only for the entire population group, with a low predictive efficacy (HR=0.601, p=0.046). Patient age, gender and treatment strategy were not associated with prognosis in any of the three groups. Detailed information is listed in Table 2 and Figure 2.

**Table 2:** Details of Cox proportional hazard model for all 3 groups

Factor	LC group			EC group			Entire population group		
	P value	HR	95% CI	P value	HR	95% CI	P value	HR	95% CI
**Age**	0.835	0.932	0.482-1.803	0.386	0.701	0.314-1.566	0.505	0.848	0.523-1.376
**Gender**	0.399	0.726	0.345-1.527	0.780	0.879	0.356-2.171	0.365	0.776	0.448-1.344
**Treatment strategy**	0.844	0.926	0.432-1.989	0.674	1.205	0.505-2.877	0.600	1.155	0.674-1.980
**Disease stage**	**0.006**	0.348	0.165-0.735	**0.001**	0.294	0.139-0.625	**<0.001**	0.346	0.209-0.574
**GM-CSF levels**	**0.006**	0.355	0.170-0.742	**<0.001**	0.202	0.082-0.497	**<0.001**	0.280	0.164-0.478
**Pre-RT IFN-*γ* levels**	0.137	0.580	0.282-1.189	0.204	0.596	0.269-1.323	**0.046**	0.601	0.365-0.991
**Integrated factor**	**0.001**	0.153	0.051-0.461	**<0.001**	0.071	0.018-0.284	**<0.001**	0.128	0.056-0.293

The table shows the details of the Cox proportional hazard model for all 3 groups. HR and 95% CI were listed. Disease stage, GM-CSF levels and the integrated factor of GM-CSF levels and pre-RT IFN-*γ* levels were independent prognostic factors. Of all 3 independent factors, the integrated factor showed the highest predictive value. Bold values stand for p<0.05.

Abbreviations: GM-CSF=granulocyte-macrophage colony stimulating factor; IFN-*γ*=Interferon-*γ*; LC=lung cancer; EC=esophageal cancer; RT=radiotherapy; HR=hazard ratio; 95% CI= 95% confidence interval.

### Correlations between GM-CSF levels and clinicopathological factors

**Figure 3:**
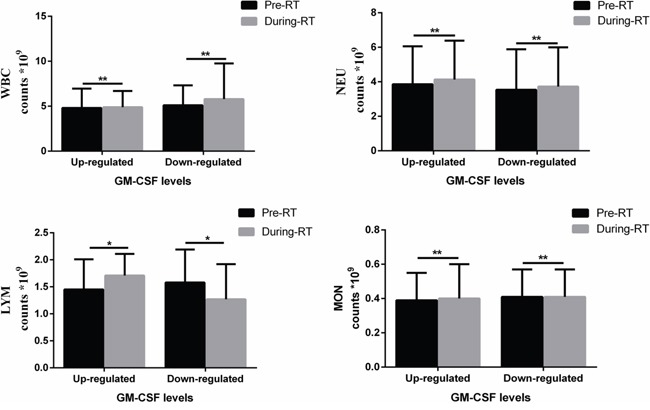
Histograms of peripheral immune cell counts in the entire population group separated by GM-CSF levels and RT LYM counts pre- and during RT showed the same change tendency with GM-CSF levels. However, WBC, NEU, and MON were not correlated with GM-CSF levels. * p<0.05.

In the LC and entire population groups, GM-CSF levels were correlated with lymphocyte (LYM) variation (LC group: p=0.038, entire population group: p=0.004). The median LYM counts were 1.56*10^9^/L (0.28-3.17*10^9^/L) in pre-RT patients and 1.405*10^9^/L (0.26-3.47*10^9^/L) in during-RT patients for the entire population group. However, proportions of CD4^+^ effector cells and regulatory T (Treg) cells were not measured. Infiltration of macrophages in irradiated patient pathological samples were not measured either. Other parameters including age, gender, disease stage, white blood cell (WBC) variation, neutrophil (NEU) variation and monocyte (MON) variation showed no correlations with GM-CSF levels. Detailed information is listed in Table 3 and Figure 3.

**Table 3:** P values for consistency of variation trend between GM-CSF and clincopathological factors

Factors	GM-CSF change		
	LC group	EC group	Entire population group
**Age**	0.164	0.846	0.371
**Gender**	0.785	0.556	0.857
**Disease stage**	0.067	0.836	0.177
**WBC change**	0.844	0.085	0.196
**NEU change**	0.673	0.22	0.26
**LYM change**	**0.038**	0.052	**0.004**
**MON change**	0.923	0.976	0.993

Of all the clincopathological factors evaluated in this study, lymphocyte change was correlated with GM-CSF levels in the LC group and entire population group. Bold values stand for p<0.05.

Abbreviations: GM-CSF=granulocyte-macrophage colony stimulating factor; LC=lung cancer; EC=esophageal cancer; WBC=white blood cell; NEU=neutrophil; LYM=lymphocyte; MON=Monocyte.

## DISCUSSION

As a basic cytokine stimulating the immune system, GM-CSF plays its therapeutic role against cancer by potentiating the antitumor effects of other molecules or treatments, such as RT [5, 18]. The interactions between this cytokine and treatment are complex, with impact on prognosis and survival being rarely investigated. In the current study we demonstrated that upregulated serum GM-CSF during RT is a positive prognostic factor for LC and EC patients. We also showed that high pre-RT IFN-*γ* also led to improved prognosis. Furthermore, this study described that elevated GM-CSF levels during RT predict longer PFS of the patients than those levels reduced during RT.

Elevated GM-CSF has been reported to act as a tumor suppressor by strengthening antigen presentation to prime tumor-specific effector T cells [7]. Therefore, fluctuations in GM-CSF levels during RT may serve as a prognostic biomarker for cancer. The potentially therapeutic combination of GM-CSF and RT is currently being tested in several clinical trials and seems to elicit an optimal immune-mediated anticancer response [19].

In this study, LYM upregulation was also tumor suppressive when combined with GM-CSF upregulation during RT, acting in cooperation with the antitumor immune cycle. However, published studies indicated that influence of RT on peripheral blood T cell subset variation is complex. A low dose (2 Gy) of whole-body irradiation significantly enhanced the proportion of CD4^+^CD25^+^Foxp3^+^ Treg cells in the periphery blood in mouse models [20]. This suggested that radiation enhanced the immunosuppressive activities of CD4^+^T effector cells and improved susceptibility of hosts to immune tolerance induction. In patients with head and neck cancers, chemoradiotherapy also resulted in decreased frequency of circulating CD4^+^ T cells (p < 0.002) and increased Treg cells (p ≤ 0.001) [21]. When optimizing the treatment strategy in murine melanoma models, 7.5 Gy/fraction also introduced the best tumor control and tumor immunity while maintaining low Treg presentation [22]. These findings warrant further studies on the interaction between T cell immune response and RT.

GM-CSF is upregulated in many cancers and studies investigating its prognostic value are controversial. For example, GM-CSF secreted by carcinomas increased recurrence and metastasis in head and neck squamous cell cancer and breast cancer [23, 24]. Furthermore, Chen *et al.* showed that high circulating GM-CSF levels were associated with poor prognosis in metastatic colorectal cancer patients [25]. On the other hand, in a study by Nebiker *et al.*, levels of GM-CSF produced by tumor cells correlated with improved survival in colorectal patients [26]. Indeed, GM-CSF has been proposed to facilitate both tumorigenic and tumor suppressive signaling cascades through differential molecular mechanisms. As a powerful activator of myeloid cells, GM-CSF is characterized by the ability to activate APCs and is widely used in cancer immunotherapy [27]. However, this cytokine is also shown to induce the expansion of myeloid-derived suppressor cells (MDSC), which have a remarkable ability to suppress T-cells [28, 29]. Our research here suggests that the antitumor effects of GM-CSF might stem from eliciting RT-induced immunogen generation, and that elevated GM-CSF levels promoted DCs and cytotoxic T LYM action, which ultimately resulted in an enhanced antitumor immune response. In the cancer immune cycle, IFN-*γ* is one of the major effector molecules, and also the major cytokine mediating the antitumor immune response [5, 7, 30]. In our current study, integration of pre-RT IFN-*γ* levels and GM-CSF levels yielded a superior predictive value than GM-CSF levels alone. This result suggests that high levels of IFN-*γ* can enhance antitumor immune effects in synergy with GM-CSF. However, since we only enrolled 126 subjects, expanding the number of patients in subsequent studies could further substantiate our conclusions and provide stronger validation for our statistical analyses. Nonetheless, our data demonstrated the prognostic vale of measuring GM-CSF levels during RT in unresectable LC and EC patients. We also provide a basis for utilizing immunotherapy combined with RT as an effective strategy for tumor treatment. However, excessive application of GM-CSF could induce immune suppression through activation of MDSC [28, 29]. Therefore, further investigations should focus on optimizing administration methods for GM-CSF under RT in large scale clinical trials.

## MATERIALS AND METHODS

### Patients

From January 2013 to October 2015, consecutive patients who received radiotherapy at Shandong Provincial Qianfoshan Hospital were enrolled in this study. This study was performed complying with the 1975 Declaration of Helsinki and was approved by the local ethics committee of Qianfoshan hospital. Each subject signed informed consent and provided blood samples at the beginning of and during RT (the median time was 32 days after beginning of RT, with a range of 30-35 days). All patients were diagnosed with EC or LC, with pathological or cytological evidence, and had at least one assessable tumor, which was included in the radiotherapy target volume. Only unresectable locally advanced or advanced cancer patients were enrolled in this study. Tumor stage was verified according to the 7th edition of the American Joint Committee on Cancer TNM classification system [31]. OS was defined from the beginning of radiotherapy to death or last follow-up. PFS was defined as the time from radiotherapy until disease progression or last follow-up. Patient follow-up data were obtained by a review of medical records and telephone.

### GM-CSF and IFN-*γ* detection

Four milliliters of blood were collected from each subject at the beginning of and during RT, and were processed within 2 h. After centrifuging at 1000 x g for 10 min, packaged serum was stored at -80 °C.

Serum GM-CSF and IFN-*γ* concentrations were measured by using a solid phase sandwich ELISA kit (Guchen Biotech, Shanghai, China). All samples were tested in duplicate following the manufacturer's instructions. The detection range was 100-280 pg/ml for GM-CSF and 80-480 pg/ml for IFN-*γ*. Blood immune cell counts were collected from medical records.

### Statistical analysis

SPSS 19.0 software (SPSS Inc., Chicago, IL, USA) was used to perform data analysis. Patients were separated into 3 groups: EC group, LC group and entire population group. GM-CSF levels were expressed as mean ± SD. The median value of pre-RT IFN-*γ* levels was defined as the cutoff value and patients were separated into two groups by that value. Upregulation and downregulation of GM-CSF and IFN-*γ* were defined by comparison of pre- and post-RT levels of these 2 cytokines for each patient. Survival curves and analysis were performed using the Kaplan-Meier method and log-rank test. To determine the independent prognostic factor, multivariate analysis was carried out using the Cox proportional hazard model, hazard ratios (HRs) and 95 % confidence intervals (95 % CIs). Correlations between GM-CSF levels and clinicopathological variables including age, gender, stage and blood immune cell count variation for all 3 groups were analyzed by applying the Pearson χ^2^ test. *P*<0.05 was considered as statistically significant. The forest plot was generated using Stata software, version 12.0 (Stata Corp, College Station, TX, USA).
